# Utility of the Comprehensive Health and Stringency Indexes in Evaluating Government Responses for Containing the Spread of COVID-19 in India: Ecological Time-Series Study

**DOI:** 10.2196/38371

**Published:** 2023-02-10

**Authors:** Kamal Kishore, Vidushi Jaswal, Anuj Kumar Pandey, Madhur Verma, Vipin Koushal

**Affiliations:** 1 Department of Biostatistics Post Graduate Institute of Medical Education and Research Chandigarh India; 2 Department of Psychology Mehr Chand Mahajan DAV College Chandigarh India; 3 Department of Health Research International Institute of Health Management Research New Delhi India; 4 Department of Community & Family Medicine All India Institute of Medical Sciences Bhatinda India

**Keywords:** COVID-19, government response, nonpharmaceutical interventions, lockdown, Comprehensive Health Index, Stringency Index, time-series modeling, ARIMA, SARIMA, Oxford COVID-19 Government Response Tracker, public health, surveillance, Oxford tracker, ecological study, health data, health policy, Bayesian information criteria

## Abstract

**Background:**

Many nations swiftly designed and executed government policies to contain the rapid rise in COVID-19 cases. Government actions can be broadly segmented as movement and mass gathering restrictions (such as travel restrictions and lockdown), public awareness (such as face covering and hand washing), emergency health care investment, and social welfare provisions (such as poor welfare schemes to distribute food and shelter). The Blavatnik School of Government, University of Oxford, tracked various policy initiatives by governments across the globe and released them as composite indices. We assessed the overall government response using the Oxford Comprehensive Health Index (CHI) and Stringency Index (SI) to combat the COVID-19 pandemic.

**Objective:**

This study aims to demonstrate the utility of CHI and SI to gauge and evaluate the government responses for containing the spread of COVID-19. We expect a significant inverse relationship between policy indices (CHI and SI) and COVID-19 severity indices (morbidity and mortality).

**Methods:**

In this ecological study, we analyzed data from 2 publicly available data sources released between March 2020 and October 2021: the Oxford Covid-19 Government Response Tracker and the World Health Organization. We used autoregressive integrated moving average (ARIMA) and seasonal ARIMA to model the data. The performance of different models was assessed using a combination of evaluation criteria: adjusted *R*^2^, root mean square error, and Bayesian information criteria.

**Results:**

implementation of policies by the government to contain the COVID-19 crises resulted in higher CHI and SI in the beginning. Although the value of CHI and SI gradually fell, they were consistently higher at values of >80% points. During the initial investigation, we found that cases per million (CPM) and deaths per million (DPM) followed the same trend. However, the final CPM and DPM models were seasonal ARIMA (3,2,1)(1,0,1) and ARIMA (1,1,1), respectively. This study does not support the hypothesis that COVID-19 severity (CPM and DPM) is associated with stringent policy measures (CHI and SI).

**Conclusions:**

Our study concludes that the policy measures (CHI and SI) do not explain the change in epidemiological indicators (CPM and DPM). The study reiterates our understanding that strict policies do not necessarily lead to better compliance but may overwhelm the overstretched physical health systems. Twenty-first–century problems thus demand 21st-century solutions. The digital ecosystem was instrumental in the timely collection, curation, cloud storage, and data communication. Thus, digital epidemiology can and should be successfully integrated into existing surveillance systems for better disease monitoring, management, and evaluation.

## Introduction

SARS-CoV-2 is contagious, capricious, and hysteric—it adversely affected the social, mental, and physical well-being of individuals and societies—from the time the first case of COVID-19 was reported from Wuhan, China, in December 2019 [1]. The World Health Organization (WHO) declared COVID-19 a pandemic in March 2020 [2]. The viral disease occurred almost a century later than the deadliest viral disease in history—the influenza of 1918-1919 [3]. Globally, COVID-19 is associated with morbidity and mortality of 458 and 6.1 million people, respectively, till March 15, 2022 [4]. However, the official numbers underestimate actual morbidity and mortality estimates—a significant reason for concern among health experts [5]. The literature points to various reasons for deflating figures: differences in disease monitoring and reporting, testing strategy, asymptomatic cases, medically unattended cases, and deaths across nations [6-8].

Many nations swiftly designed and executed government policies to contain the rapid rise of COVID-19 cases [9]. Government actions can be broadly categorized as movement and mass gathering restrictions (such as travel restrictions and lockdown), public awareness (such as face masking and hand washing), emergency health care investment, and social welfare provisions (such as poor welfare scheme and providing food and shelter to migrant workers) [10]. However, the intermix of culture, communication, society, religion, politics, socioeconomic disparities, and education plays a dominant role in successfully implementing government policies [11,12]. Despite aggressive interventions by the Government of India (GOI), mass migration and verbal and physical abuse against health care providers were perplexing during the COVID-19 crisis [12-14]. These problems indicate that implementing policies to contain the public health crisis is one part of the puzzle, and understanding, adherence, and participation by citizens to make it a success is the other. Thus, a mismatch and mistrust between the public and policy makers will determine how the crises unfolds [15,16]. Despite these constraints, the principal priority was to contain the pandemic and mitigate future waves.

Traditional surveillance systems are robust and well-developed in many countries, but these were insufficient during the COVID-19 pandemic. Prominent challenges are that physical networks are extremely slow to upgrade (such as surveillance and physical structure), resource-intensive (in terms of finances and personnel), and involve bureaucratic hurdles (such as permissions and administrative authority). An emergency or crisis demands agile and innovative solutions. Internet technology can supplement the already existing traditional structure. The technological innovation of the internet and digital devices has fundamentally reshaped how people seek information and adhere to guidelines [17,18]. Therefore, blending existing surveillance systems with a digital ecosystem is ideal for harnessing and optimizing their untapped potential. Technology, medical development, health infrastructure, and lifestyle are pivotal in combating public health crises.

Many studies have highlighted the importance of nonpharmaceutical Interventions (NPIs) in containing the COVID-19 crisis [19-22]. Internet and digital devices played a critical role in screening, surveillance, monitoring spread, sharing, and managing data [23]. However, most of the studies focused on mobility during the current crisis. Mobility is vital, but many other essential indicators of disease spread exist. The Oxford tracker keeps track of various policy initiatives to mitigate COVID-19 and releases them as Comprehensive Health Index (CHI) and Stringency Index (SI) data [24]. CHI and SI are composite indicators covering the various aspects of NPIs; these indicators provide a systematic approach to understanding how the government has responded to mitigate disease spread over a period. We aimed to assess the utility of CHI and SI—innovative measures of the University of Oxford—to gauge and evaluate government responses for containing the spread of COVID-19. We hypothesize that there is a significant inverse relationship between policy indices (CHI and SI) and COVID-19 severity indices (morbidity and mortality). The analyses hope to inform and better prepare the experts to face and refine epidemiological indicators for future contagious disease crises.

## Methods

### Study Design

An ecological time-series study design was adopted, using secondary data.

### Study Period

The first case of COVID-19 in India was reported from Kerala on January 27, 2020 [25]. The GOI responded to the COVID-19 threat by issuing travel advisories and screening at airports in January and later restricting international travel from March 2020 onward [26,27]. As the cases in India started escalating, the GOI called for a *Janata* curfew on March 23, 2020, and implemented a national lockdown from March 24 to June 7, 2020, for 75 days [28]. The GOI started phased unlocking from June 8, 2020, onward [28]. COVID-19 and SI data for India are available from January 21, 2020, to March 2022. This study used data from March 4, 2020 (when India crossed 10 cumulative cases), to October 24, 2021.

### Data Sources

We used 2 publicly available data sources from the Oxford Covid-19 Government Response Tracker (OxCGRT) [29] and the WHO [4] licensed under the Creative Commons Attribution (CC BY) standard.

#### COVID-19 Data

The WHO collects the numbers of confirmed COVID-19 cases and deaths through official communications under the International Health Regulations of 2005. WHO experts monitor the official ministries of health’s websites and social media accounts for data validation. The data set, after curation, is made available as a .csv file and can be downloaded from the website [24].

#### CHI and SI Data

The OxCGRT for containment and closure and health data set evaluates the government response to containing the COVID-19 crisis. The OxCGRT collected 23 indicators to capture government policies related to closure and containment, health, and economics for more than 180 countries since January 1, 2020. The policy indicators are captured on an ordinal or numerical scale to describe each category’s degree or the strength of the government response. Each ordinal indicator is transformed to a value between 0 and 100 per the formula given in the codebook [25]. Finally, to gauge government performance, individual indicators are aggregated and published in 5 composite indices: Government Response Index, Containment and Health Index, SI, Economic Support Index, and Legacy Stringency Index. The CHI uses 14 indicators and SI uses 9 indicators out of 23. The value for composite indices on any given day represents the aggregated average of each indicator for the day. The composite indices report a number between 0 and 100 that reflects the overall stringency of the government’s response. A higher index indicates a higher overall response level. Table 1 shows details regarding individual indicators used to calculate the CHI and SI, both of which are updated regularly. The indicators are reported for each day that a policy was implemented (not on the day it was announced). A continuously updated OxCGRT index provides comparable information regarding various countries’ policy measures. Many indicators have another flag variable to imply whether they are targeted (applying only to a subregion of a jurisdiction or to a specific sector) or general. The OxCGRT uses simple, additive, and unweighted indices that are easier to interpret. The missing value contributes a zero to the index. Multimedia Appendix 1 demonstrates the steps to download the OxCGRT data sets. Details of all the indicators and calculations for the composite index are provided in the working paper entitled, “Variation in government responses to COVID-19” [30].

**Table 1:** Details regarding individual and composite Oxford Covid-19 Government Response Tracker indicators.

ID		Policy measures	Scale	Score, maximum (range)	Stringency Index	Comprehensive Health Index	Flag^a^
**Containment and closure**							
	C1	School closing	Ordinal	3 (0-3)	✓	✓	Yes=1
	C2	Workplace closing	Ordinal	3 (0-3)	✓	✓	Yes=1
	C3	Cancel public event	Ordinal	2 (0-2)	✓	✓	Yes=1
	C4	Restriction on gathering size	Ordinal	4 (0-4)	✓	✓	Yes=1
	C5	Close public transport	Ordinal	2 (0-2)	✓	✓	Yes=1
	C6	Stay-at-home requirements	Ordinal	3 (0-3)	✓	✓	Yes=1
	C7	Restriction on internal movement	Ordinal	2 (0-2)	✓	✓	Yes=1
	C8	Restriction on international travel	Ordinal	4 (0-4)	✓	✓	No=0
**Health systems**							
	H1	Public information campaign	Ordinal	2 (0-2)	✓	✓	Yes=1
	H2	Testing policy	Ordinal	3 (0-3)		✓	No=0
	H3	Contact tracing	Ordinal	2 (0-2)		✓	No=0
	H6	Facial covering	Ordinal	4 (0-4)		✓	Yes=1
	H7	Vaccination policy	Ordinal	5 (0-5)		✓	Yes=1
	H8	Protection of older individuals	Ordinal	3 (0-3)		✓	Yes=1

^a^A value of 0 indicates that the policy is targeted to a subregion.

### Study Variables

#### Dependent Variables

The data about morbidity and mortality were downloaded in the .csv file and reported using standardized metrics such as cases per million (CPM) and deaths per million (DPM). CPM and DPM are valuable indicators for intergeographical comparisons—the same metrics were calculated and reported using standard formulae provided by the WHO. Multimedia Appendix 2 provides details about the 7-day moving average, CPM, and DPM.

#### Independent Variables

Government policies and timing are vital indicators of morbidity and mortality related to disease during and after the crisis. Therefore, we used time, CHI, and SI, which are cumulative nonweighted indices of government policies as predictor variables. Table 1 provides detail of both CHI and SI.

### Data Analysis

#### Data Processing and Summarization

We retained originally downloaded data sets of COVID-19 and OxCGRT for record and referral. Subsequently, a copy of each original data set was generated to clean, code, and analyze. Data cleaning in both files involved renaming, relabeling, and removing of undesired variables. Subsequently, we used the *date* variable to merge and prepare a final data set for analysis. The raw day–wise data were smoothed using a 7-day moving average that was subsequently used to calculate and report CPM and DPM. The morbidity indicator in the country was summarized using frequency and percentage increase. Initially, time-series plots were used to gauge the disease burden. Subsequently, a dual-axis chart was used to visualize the pattern between disease burden and policy indicators. The descriptive tables and graphs were prepared using Excel (Microsoft Corp).

#### Time-Series Modeling

The daily SARS-CoV-2 data follow the characteristics of the time series. As an initial step, we carefully inspected the daily incidence (morbidity and mortality), descriptive statistics (mean and variance), and seasonality (weekly and periodicity). We subsequently used second-order differencing for CPM and first-order differencing for DPM to make them stationary, which is an essential requirement to apply time-series analysis [31]. The twin advantage of simple structure and immediate applicability of autoregressive integrated moving average (ARIMA) and seasonal ARIMA (SARIMA) made them lucrative for analyzing the current study data set [32]. ARIMA considers only the past values for prediction, whereas SARIMA also considers the seasonality patterns, making it a more robust algorithm for prediction. We plotted autocorrelation function (ACF) and partial ACF (PACF) values to assess autoregressive and moving average components of the ARIMA and SARIMA.

#### Model Construction and Comparison

We could not finalize the order of autoregressive and moving average components from ACF and PACF plots. Therefore, considering the Expert Modeler and Box-Jenkins 5-step methodology (describe data, identify model, estimate parameters, diagnosis check, and forecasting), we relied on an expert modeler for an initial model from multiple candidate models. Subsequently, we used the Box-Jenkins technique that gives the flexibility to customize and attain the final model [33]. We used the date (time) as an explanatory variable in the initial model. Subsequently, CHI or SI and time were added to an expanded model to build and evaluate the models. The performance of different models was assessed using a combination of evaluation criteria: adjusted *R*^2^, root mean square error, and Bayesian information criteria (BIC). After attaining the final model, we performed the diagnosis check to validate model assumptions. Multimedia Appendix 3 elucidates the step-by-step approach to attain the final model. We used a 2-tailed *P* value of <.05 to declare statistical significance. The time-series analysis of data was performed using SPSS (version 23; IBM Corp).

### Ethical Considerations

Ethical clearance for the study was obtained from the institutional review board of the Postgraduate Institute of Medical Education and Research, Chandigarh, India (vide letter INT/IEC/2020/SPL–1594).

## Results

### Disease Burden

**Figure 1:**
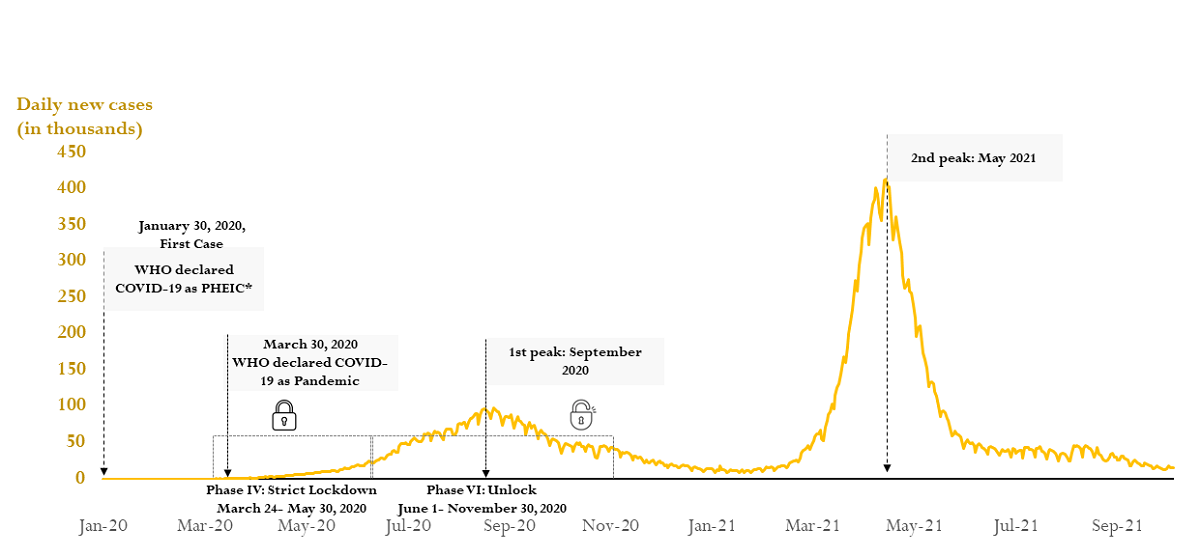
A line graph (epidemic curve) showing daily new COVID-19 cases in India. Adapted from the World Health Organization's (WHO's) Coronavirus Dashboard [4]. PHEIC: public health emergency of international concern.

The first case of COVID-19 was detected in India on January 27, 2020. It took 46 days for the infection to spread among 100 people. India had 60 COVID-19–infected individuals on March 11, 2020, when the WHO declared COVID-19 a pandemic. True to its nature, COVID-19 started spreading rapidly, and it took only 15 days to reach 1000 cases from 100. Public health experts and policy makers were on high guard after GOI notification—a fact also reflected by the SI and CHI. Table 2 provides details about different milestones regarding the rise in disease burden. The increase in cases followed a linear trajectory during the lockdown stage. After unlocking, India achieved its first peak in September 2020, which was almost 100,000 cases per day. Subsequently, the second peak in May 2021 was characterized by a maximum caseload of ~400,000 cases per day. Figure 1 shows the change in India’s disease burden from January 2020 to October 2021.

**Table 2:** Different milestones in the rise of the COVID-19 burden in India.

Date	Lockdown phases	Cumulative COVID-19 cases in India, n	Time from the report of the first COVID-19 case in India	Stringency index, median (range)	Comprehensive Health Index, median (range)
January 27, 2020	No	1	Frist case	—^a^	—
March 4, 2020	No	10	n=35 (1 month)	10.2 (10.2-10.2)	17.3 (13.7-17.3)
March 15, 2020	No	100	n=46 (1.5 months)	29.4 (26.9-38.9)	29.6 (27.9-35.7)
March 30, 2020	Phase 1: March 25-April 14, 2020	1000	n=61 (2 months)	81.7 (48.2-100)	66.8 (41.7-80.9)
April 14, 2020	Phase 2: April 15-May 3, 2020	10,000	n=76 (2.5 months)	100 (100-100)	86.2 (80.9-91.9)
May 19, 2020	Phase 4: May 18-May 31, 2020	100,000	n=111 (4 months)	90.3 (81.9-100)	84.1 (76.8-91.9)
July 17, 2020	Unlock 2.0: July 1-July 31, 2020	1,000,000	n=170 (5.5 months)	86.4 (81.9-87.5)	74.9 (74.4-76.8)
December 19, 2020	Partial	10,000,000	n=325 (10.5 months)	75.9 (61.6-87.5)	68.8 (61.3-74.4)
May 4, 2021	Partial	20,000,000	n=461 (15 months)	65.6 (57.9-74.5)	71.3 (66.1-81.1)
June 23, 2021	Partial	30,000,000	n=547 (18 months)	81.1 (73.6-81.9)	79.4 (74.5-79.8)

^a^Not determined.

### Epidemiological and Policy Indicators

**Figure 2:**
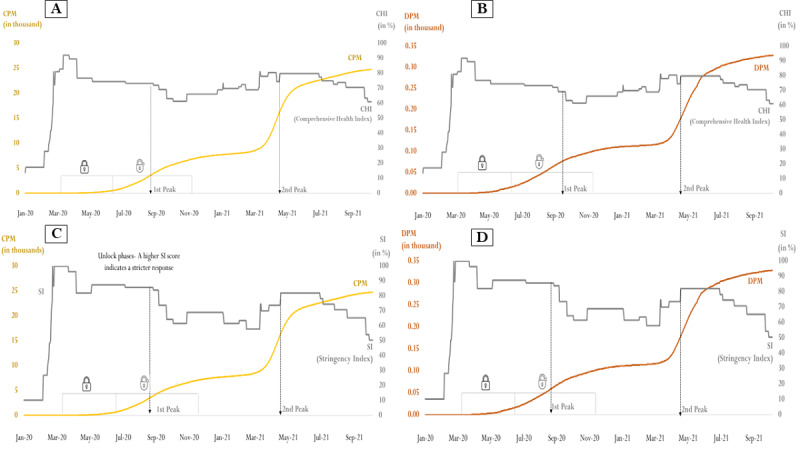
Line graphs depicting changes in the Comprehensive Health Index or Stringency Index (SI) versus cases per million (CPM) or deaths per million (DPM) for SARS-CoV-2 in India. (A) Change in the Comprehensive Health Index and CPM, (B) change in the Comprehensive Health Index and DPM, (C) change in the Stringency Index (SI) and CPM, and (D) change in the SI and DPM. Data obtained from the World Health Organization's Coronavirus Dashboard [4] and from GitHub [28].

The strict implementation of policies by the government to contain the COVID-19 crisis led to a very high value of CHI and SI in the beginning. Figure 2A shows that the CPM was near the x-axis from the beginning till the end of the fourth lockdown. Subsequently, CHI reduced gradually; in contrast, the CPM swiftly increased after the GOI initiated unlocking from July 2020. Figure 2B shows that the DPM also increased steadily with time—the highest during the unlock phase. Although the CHI and SI reduced marginally, there were spikes during the first and second peaks. The CPM and DPM also displayed similar increasing trends with the SI. Figures 2C and 2D depict CPM and DPM changes with SI over time.

### Time-Series Modeling

Figures 1 and 2A-2D show that both positive (symptomatic) and deaths (mortality) were nonstationary time series. On the surface, the CPM and DPM followed the same trend. However, the final CPM and DPM model were SARIMA (3,2,1)(1,0,1) and ARIMA (1,1,1), respectively. Multimedia Appendix 3 provides details regarding the steps involved in obtaining the final models. There was no improvement in model performance for CPM: the BIC values for both time (BIC 2.42) and the expanded model (BIC 2.46) were almost the same. Similarly, the BIC values for both time (BIC –1.35) and the expanded model (BIC –1.34) models for DPM were the same. Despite the appearance of an inverse relationship between COVID-19 and policy indicators, neither CHI nor SI were significant predictors of CPM and DPM. Table 3 displays the final model’s output and performance indicators: BIC, stationary *R*^2^, and root mean square error.

**Table 3:** The output of the final model for COVID-19 cases per million and deaths per million in India from March 2020 to October 2021.

Model and parameter		Estimate	*P* value	Bayesian information criteria		Stationary *R*^2^	Root mean square error	
**Seasonal autoregressive integrated moving average (3,2,1)(1,0,1)**					2.42	0.62		3.22
	AR1	0.67	<.001					
	AR2	0.12	.02					
	AR3	0.10	.04					
	MA1	0.73	<.001					
	SAR1	0.86	<.001					
	SMA1	0.25	<.001					
**Autoregressive integrated moving average (1,1,1)**					–1.35	0.64		0.50
	AR1	0.99	<.001					
	MA1	0.82	<.001					

## Discussion

### Principal Findings

We used the CHI and SI data from the Blavatnik School of Government, University of Oxford, response tracker to evaluate and model COVID-19 morbidity and mortality. There are specific critical findings in our study. COVID-19 displayed the typical characteristic of a highly contagious disease that spread rapidly after a slow start. At the beginning of the pandemic in India, cases were geographically scattered and scarce; thus, the CHI and SI were low as neither the GOI issued any guidelines nor did the people adhere to preventive behaviors. There was a sharp increase in cases after unlocking, and India witnessed 2-clear and visible peaks in September 2020 and May 2021; this was in line with a temporary increase in CHI and SI during peaks. The value of CHI and SI gradually decreased; similarly, however, these were consistently higher at values of >80% points.

For intercountry comparisons, the CPM and DPM were calculated and reported, since these are better measures than the raw number of cases. The CPM and DPM linearly increased when stringency was high during the lockdowns and rapidly increased after the government lifted the restrictions. Despite initial indication, both CHI and SI were (rather surprisingly) not significant predictors of COVID-19 morbidity and mortality.

People’s participation is vital to the success of public health interventions. Thus, the GOI tested people’s moods and sentiments with the *Janata Curfew* (self-restricted curfew) before implementing strict public health measures. After the success of the Janata curfew, the GOI enforced a nationwide lockdown to close schools, parks, transport, offices, and borders to contain the COVID-19 crisis. The daily incidence was relatively flat during the entire lockdown period; the strict response to epidemic control measures reduced daily incidence [26]. However, social control measures mitigate contagious diseases; they do not eradicate them [27,28]. The rate of disease spread increased rapidly after the unlocking process began. Despite the nationwide lockdown, significant intra- and interstate variation was a cause of concern—the majority (~76%) of cases were reported from 10 out of 36 states and union territories in India [29]; the disparity can be partially attributed to constitutional provisions besides factors such as mobility, urbanization, and population density. Moreover, while the central government has the power to make public health laws, the state government develops infrastructure and executes public health policy because health care and hospitals are subject matters in the state list [4].

NPIs such as tests, contact tracing, masking, social distancing, mobility, hygiene, and vaccination form the backbone for controlling contagious diseases. However, in the absence of vaccination, strategic implementation of NPIs helps mitigate the rapid spread of COVID-19 [13-16]. The CHI and SI are more comprehensive than specific mobility indicators—these cover various aspects of NPIs. The CHI and SI attain very high values, and the results are in parallel with those of Ma et al [34], which indicate that countries that implemented nationwide lockdowns in March took strict measures [26]. The high value of CHI and SI during the lockdown reflects public health experts’ and policy makers’ comprehensive and dynamic responses to contain the COVID-19 crisis. Government-enforced lockdowns are significant contributors to restrictions on activities. People also respond to contagious threats by restricting socializing and travelling [35]. Overall, CHI and SI reflected high stringency; this further increased during the peak. Initial investigation reveals a distinct inverse relationship between restriction indicators (CHI and SI indices) and disease burden (morbidity and mortality); this relationship was explicit as the unlocking phase began.

We fitted a time-series model to investigate the relationship between policy and disease indicators. The time-series model with dates explained more than 60% of variability for both CPM and DPM models. The initial investigation indicated the relationship between predictors (CHI and SI) and disease burden (CPM and DPM). However, the expanded inferential models do not increase the models’ performances—neither CHI nor SI contributed significantly explaining the change in CPM and DPM. In other words, stringency measures such as CHI and SI do not explain the change in both COVID-19 morbidity and mortality. The results of our study are in contrast to those of other studies [26,30,31]. However, it may be crucial to note that strict implementation of policies does not necessarily lead to strict compliance [32,33]. A thorough inspection of CHI and SI indicates the government’s proactiveness on different indicators compared to participation. India saw the mass movement of migrant workers [34,36] and multiple attacks on health care workers during the lockdowns [34,37]. Further, COVID-19 was a relatively urban phenomenon; most of India is rural.

### Strengths and Limitations

A significant strength of our study is that this is the first study to investigate the relationship between policy indicators CHI and SI and epidemiological indicators CPM and DPM. It is the first pan-India study that has used WHO and OxCGRT data to quantify and model COVID-19 transmission. Our study is different from other studies focused on NPIs including mobility or SI but not CHI.

However, a significant limitation of this study, and perhaps most digital epidemiological studies, is the validity and reliability of the data. As already highlighted, CHI and SI are proxy measures that do not reflect strict compliance with the implementation of policies. There may be an inherent bias or reporting error in composite CHI and SI indices at the country level due to within and between heterogeneity at the state and union territory levels. The unweighted indices (CHI and SI) are easy to interpret but make strong assumptions, and a user may obtain different results when using weighted indices. Lastly, correct reporting of the daily incidence of COVID-19 cases depends on a country’s accuracy and testing capacity.

### Future Work

Countries with vast geographical and administrative regions may differ significantly in implementing policies. Therefore, further research is required to develop and validate the metrics to identify whether country- or county-level (state and union territory) metrics are needed. The capacity to test, trace, and treat varies from country to country. Do disease morbidity and mortality reporting reflect the actual scenario? What logical steps and weightage must be given to each region is also a perplexing problem vouching for researchers’ attention. The media plays a vital role in containing public health crises. However, did it aid in signaling by motivating people to adhere to guidelines, or has it added to noise by creating panic among the public? Lastly, are OxCGRT indicators enough to capture people’s participation and policy makers’ execution or do they require appropriate weightage? The aforementioned issues invite attention from health experts and policy makers.

### Conclusions and Recommendations

Our study concludes that the policy measures (CHI and SI) do not explain the change in epidemiological indicators (CPM and DPM). The study reiterates our understanding that strict policies do not necessarily lead to better compliance but may overwhelm the overstretched physical health systems. Twenty-first–century problems thus demand 21st-century solutions. The digital ecosystem was instrumental in the timely collection, curation, cloud storage, and data communication. Thus, digital epidemiology can and should be successfully integrated into existing surveillance systems for better disease monitoring, management, and evaluation. An OxCGRT policy metric is a novel innovation to assess government actions during the epidemic, which have the potential for future use and refinement. Therefore, policy makers, public health experts, and programmers must start collaborating to design a hybrid health system that can borrow from the strengths of the existing physical surveillance system and the ever-expanding digital ecosystem.

## Appendix

Multimedia Appendix 1 Summary of Steps to download the OxCGRT datasets.

Multimedia Appendix 2

Multimedia Appendix 3 Details of the steps to obtain final models.

## Abbreviations

ACF: autocorrelation function
ARIMA: autoregressive integrated moving average
BIC: Bayesian information criteria
CHI: Comprehensive Health Index
CPM: cases per million
DPM: deaths per million
GOI: Government of India
NPI: nonpharmaceutical intervention
OxCGRT: Oxford Covid-19 Government Response Tracker
PACF: partial autocorrelation function
SARIMA: seasonal autoregressive integrated moving average
SI: Stringency Index
WHO: World Health Organization
