# Supplementary material for: Utility of the Comprehensive Health and Stringency Indexes in Evaluating Government Responses for Containing the Spread of COVID-19 in India: Ecological Time-Series Study
Source: JMIR Public Health Surveill. 2023 Feb 10;9:e38371. doi: 10.2196/38371 (PMC9924057; doi:10.2196/38371)
Supplement: Multimedia Appendix 1 [file publichealth_v9i1e38371_app1.docx]

**Appendix 1: Steps to download the OxCGRT datasets**

| **Step 1: Go to the Oxford** [**COVID-19 Government Response Tracker**](https://www.bsg.ox.ac.uk/research/research-projects/covid-19-government-response-tracker) **website** | **Step 2: Scroll down to our data** |
| --- | --- |
| **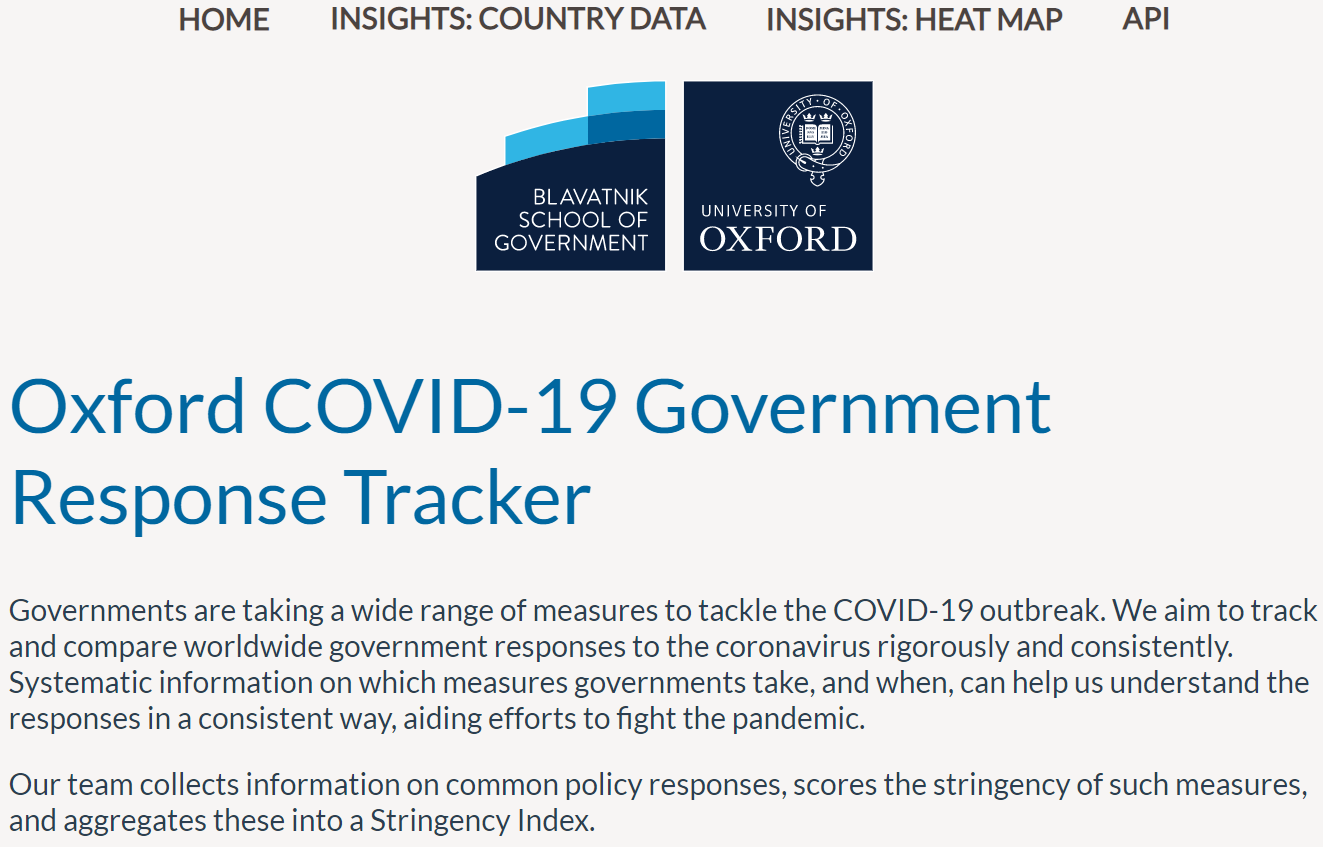** | **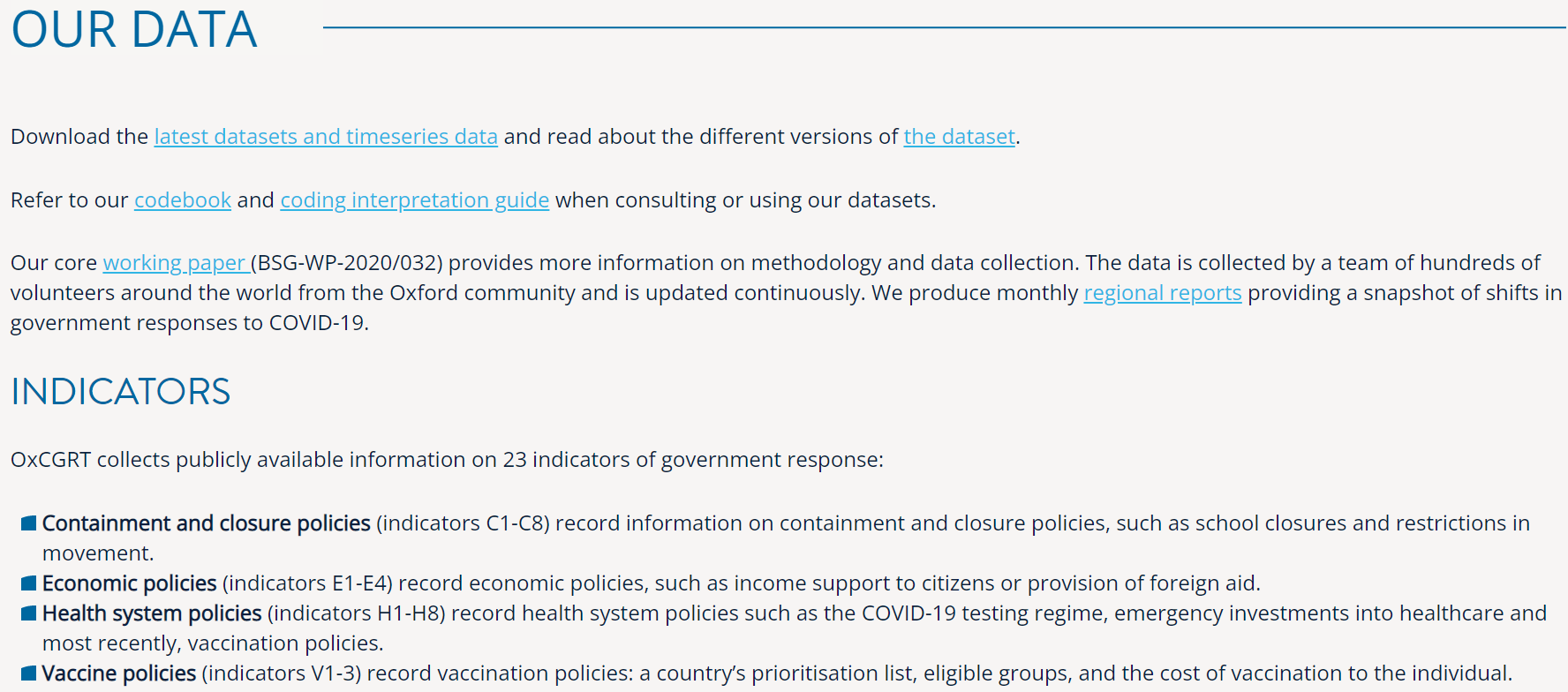**  Click on the links to data |
| **Step 3: Link will go to GitHub data repository** | **Step 4: Downloading data from repository** |
| **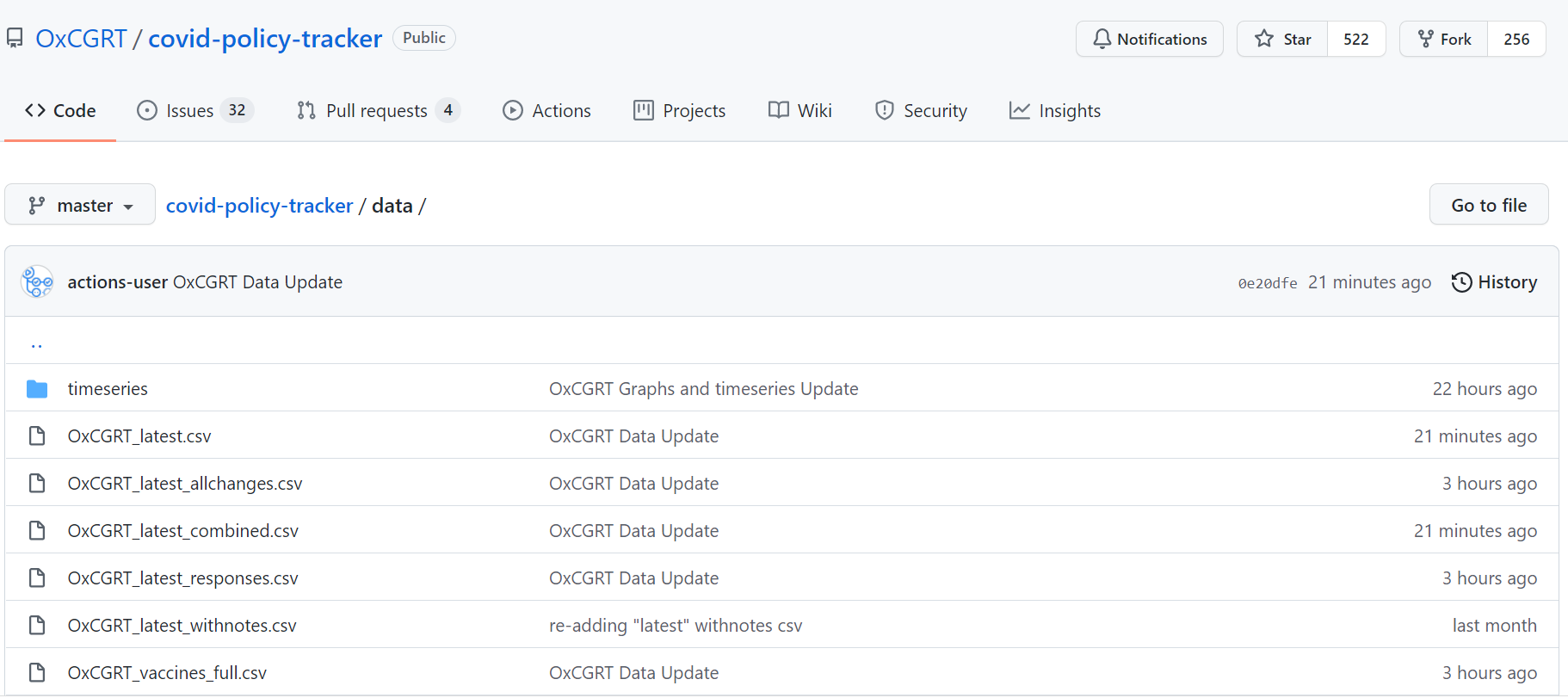**  Select latest CSV file to download data | **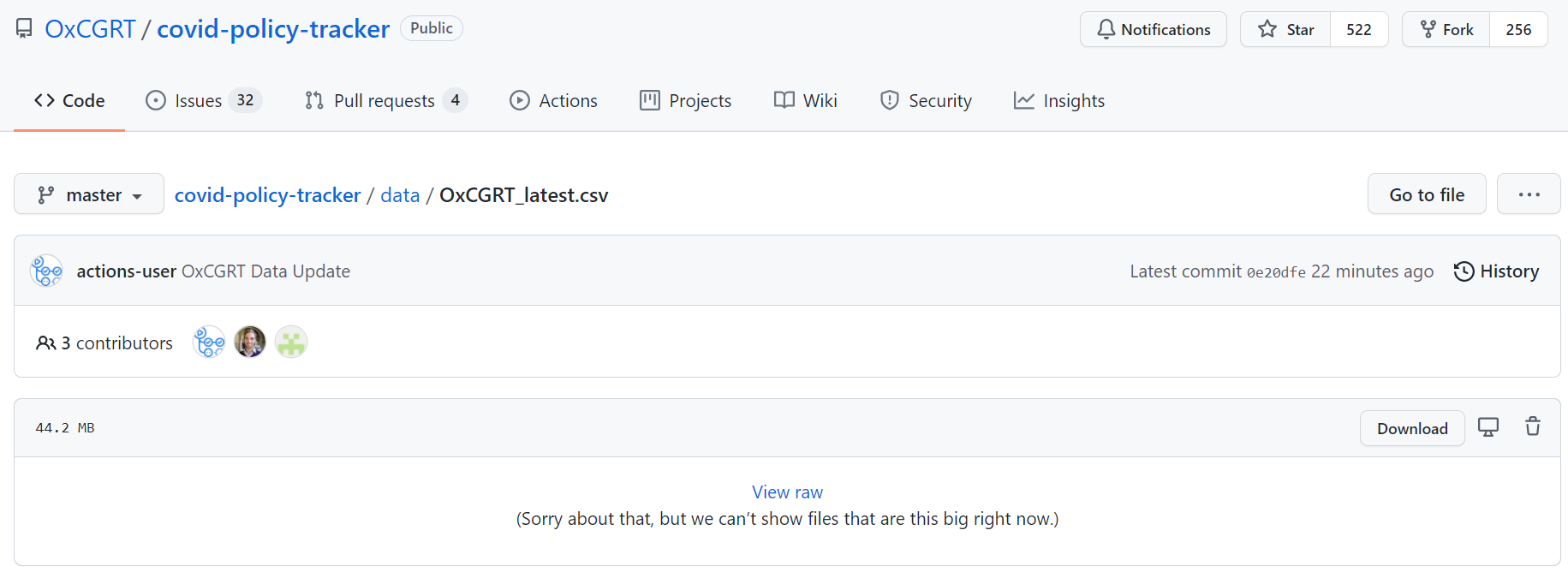**  Click View Data and use Ctrl/Cmd+s to download data |
