# Supplementary material for: Utility of the Comprehensive Health and Stringency Indexes in Evaluating Government Responses for Containing the Spread of COVID-19 in India: Ecological Time-Series Study
Source: JMIR Public Health Surveill. 2023 Feb 10;9:e38371. doi: 10.2196/38371 (PMC9924057; doi:10.2196/38371)
Supplement: Multimedia Appendix 2 [file publichealth_v9i1e38371_app2.docx]

**Appendix 2: Details regarding 7-day moving average, cases per Million population (CPM) and Deaths per Million population (DPM).**

**Moving Average**

MA is a statistical calculation process that creates a dataset from original data by averaging the subset of data. Researchers can calculate and report three, five, seven days, or any other moving average as per requirements. However, the seven-day MA is most prevalent in health settings. The 7-day MA is obtained by taking the arithmetic mean of 1^st^ seven observations. Subsequent values of MA are calculated using successive values by removing 1^st^ observation from the series and adding next to the last value of the series till the end of all the values. Here is an example:

Frequency of cases: 1, 3, 5, 4, 20, 2, 7, 9,11

7-day MA: -, -, -, -, -, -, 6, 7.14, 8.28

**Death per million (DPM)**

As per WHO, DPM is a valuable indicator of disease mitigation and case management–the most helpful reflector of a country's health system. It tells whether the problem of the epidemic is increasing or decreasing. It is a valuable indicator for inter-geographical comparisons. Mortality per million is the ratio of mortality due to disease and the total population of an area.

DPM = Mortality due to the disease in the India/Total projected population of the India * 10^6^

**Cases per million (CPM)**

The number of cases in the countries depends on the nations' population–generally, more the people higher are the cases and vice-versa. A similar argument is valid for the various administrative and political areas of different sizes and populations. Therefore, simple calculation and reporting of positives are not ideal for comparison within and between nations. Case per million (CPM) is a valuable standard for comparison. The CPM is the ratio of cases to the total population of a place.

CPM = frequency of Cases in India/Total projected population of India*10^6^
