# Supplementary material for: Utility of the Comprehensive Health and Stringency Indexes in Evaluating Government Responses for Containing the Spread of COVID-19 in India: Ecological Time-Series Study
Source: JMIR Public Health Surveill. 2023 Feb 10;9:e38371. doi: 10.2196/38371 (PMC9924057; doi:10.2196/38371)
Supplement: Multimedia Appendix 3 [file publichealth_v9i1e38371_app3.docx]

**Appendix 3: Details of the steps to obtain final models**

***Step 1*:** Detecting Non-stationarity in the Data

We started with making time series plot for both CPM and DPM. Figures 1(a) and 1(b) of CPM and DPM, respectively, indicate that neither cases nor death is stationary over time.

| 1(a) Time series plot for CPM | 1(b) Time Series Plot for DPM |
| --- | --- |
| 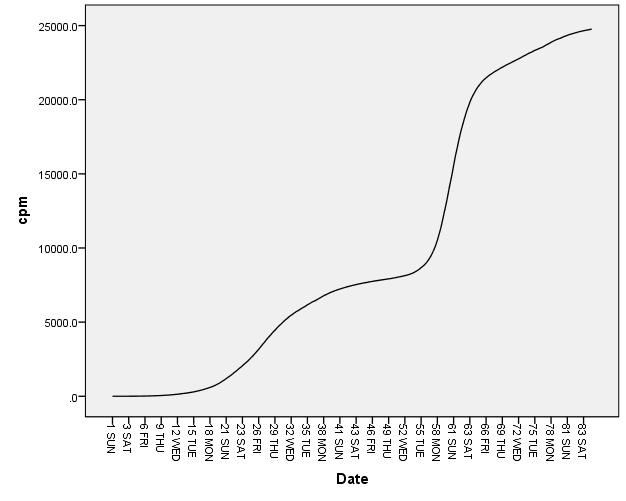 | 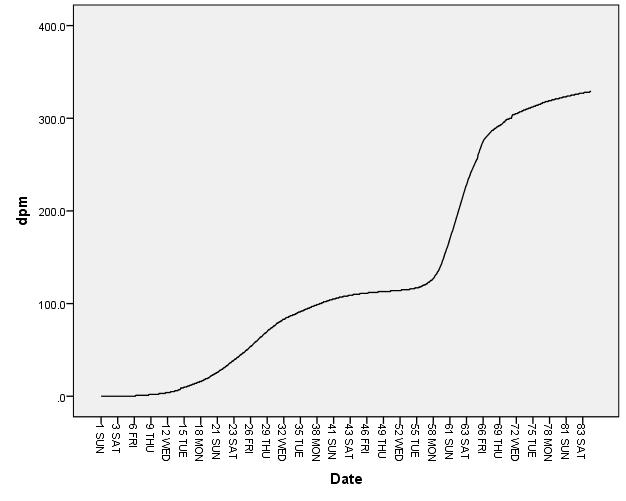 |

**Step 2:** **Transforming non-stationarity data to stationarity**

The 1^st^ order differencing did not produce stationarity time series for cases. Thus, 2^nd^ order differencing was used. The stationarity time series for death was obtained with 1^st^ order differencing. Figures 2(a) and 2(b) display that differencing of orders 2 and 1 for cases and deaths were used, respectively. Fig 2(c) and 2(d) display the ACF and PACF plot for frequency of cases–both plots indicate seasonality as there is a significant lag at 7, 14 and 21 days. Thus we selected seasonal differencing (D) of order 1.

| 2(a): Stationary time series for case | 2(b): Stationary time series for death |
| --- | --- |
| 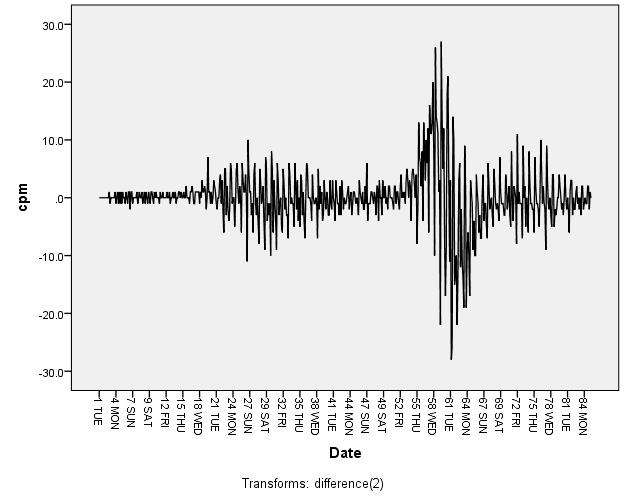 | 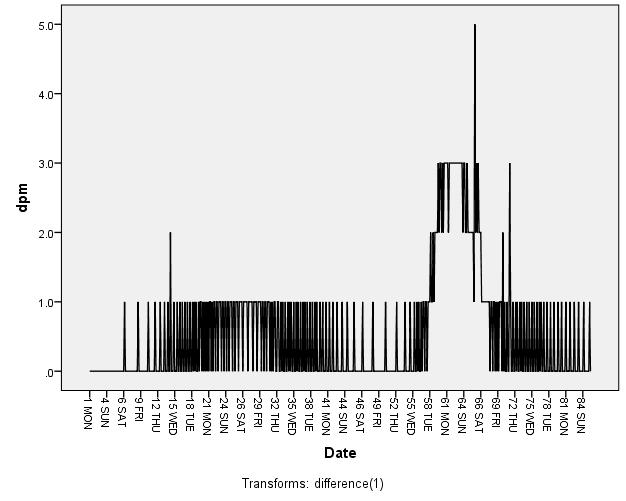 |
| 2(c): ACF plot for cases | 2(d): PACF plot for cases |
| 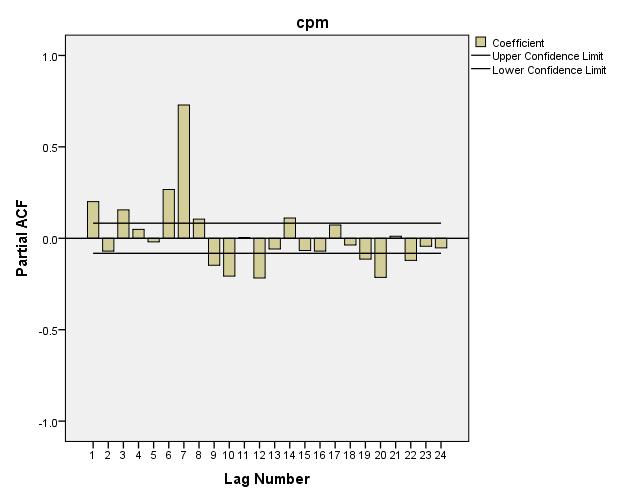 | 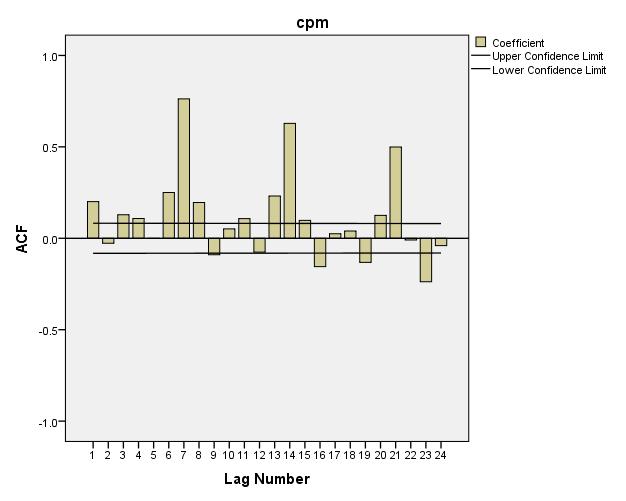 |

**Step 3: Model Identification**

1. *Identifying p, P, q and Q for CPM*

To identify the value of p, P, q and Q for frequency of cases, we again made PACF and ACF plots with d=2 and D=1. PACF plot in figure 3(a) depicts significant autocorrelation at lag 1,3, and 6 that indicates the value of p to be 1, 3, and 6. There is also a significant correlation at lag 7 and 14, but lag 7 is significantly more than lag 14. The lag indicates the most probable value of P=1, followed by P=0.

Figure 3(b) depict significant autocorrelation at lag 1, 3, 5 and 6 days that indicate q to be 1, 3, 5 and 6. There is also a significant autocorrelation at lag 7 that show Q=1.

1. *Identifying p, P, q and Q for DPM*

PACF plot in 3(c) depicts significant autocorrelation at lag 1, 3 and 6, which indicate p to be 1,3 and 6. The significant autocorrelation at lag 7 and 14 indicate either P=1 or P=0.

Figure 3 (d) depict a significant correlation at lag 1, 3, 4 and 6 days that depict q to be 1, 3, 4 and 6. Whereas significant autocorrelation at lag 7, 14 and 21 indicate Q=1.

| 3(a): PACF model for CPM | 3(b): ACF model for CPM |
| --- | --- |
| 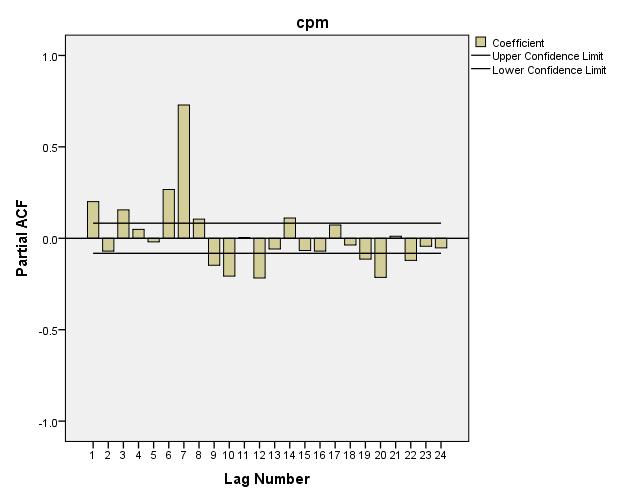 | 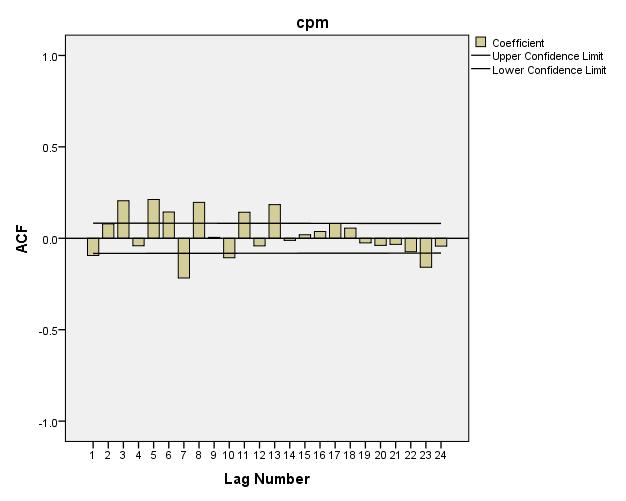 |
| 3(a): PACF model for DPM | 3(b): ACF model for DPM |
| 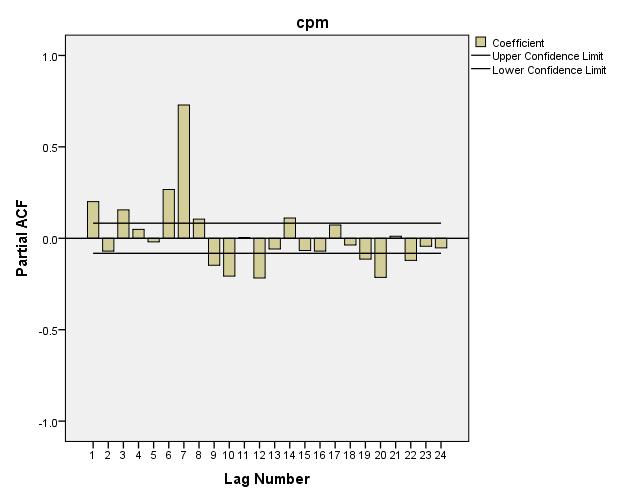 | 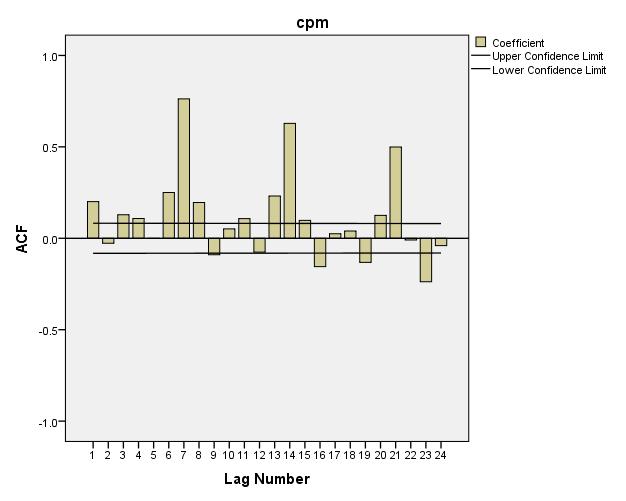 |

**Step 4: Parameter Estimation**

1. *Model for CPM*

To obtain the initial model from potentially many models, we opted to run an expert modeller in SPSS that automatically select the model's parameters. The expert modeller gave us SARIMA(3,2,6)(1,0,1) as the initial model to begin. We did not want to propose to overfit model – the same is usually inefficient in generalization to realistic settings. Considering parsimony and **George E. P. Box's** approach to model building, *“All Models are Wrong, but some are useful.”* We compared the various models before selecting the final models. The comparative indices of multiple models are given in table 1.

Table 1: Comparative performances of various SARIMA models for CPM

| **Model** | **Stationary**  **R-squared** | **RMSE** | **Normalized BIC** |
| --- | --- | --- | --- |
|  |  |  |  |
| SARIMA(3,2,6)(1,0,1) | 0.62 | 3.19 | 2.38 |
| SARIMA(3,2,6)(1,1,1) | 0.27 | 3.11 | 2.40 |
| SARIMA(3,2,5)(1,0,1) | 0.64 | 3.17 | 2.43 |
| SARIMA(3,2,5)(0,0,1) | 0.46 | 3.84 | 2.80 |
| SARIMA(3,2,5)(1,0,0) | 0.63 | 3.19 | 2.43 |
| SARIMA(3,2,4)(1,0,1) | 0.64 | 3.15 | 2.40 |
| SARIMA(3,2,3)(1,0,1) | 0.62 | 3.21 | 2.43 |
| **SARIMA(3,2,2)(1,0,1)*** | **0.62** | **3.22** | **2.42** |
| SARIMA(3,2,1)(1,0,1) | 0.62 | 3.22 | 2.42 |
| SARIMA(2,2,2)(1,0,1) | 0.61 | 3.28 | 2.45 |
| SARIMA(1,2,1)(1,0,1) | 0.59 | 3.35 | 2.47 |
| SARIMA(1,2,1)(0,1,0) | 0.01 | 3.59 | 2.59 |
|  |  |  |  |

** -> Final Selected Model*

1. *Model for DPM*

We obtained a holt model with expert modeler. Holt's model is similar to an ARIMA(0,2,2) model. Thus we ran custom ARIMA(0,2,2) as the initial model. The comparative indices of different models are given in table 2.

Table 2: Comparative performances of various ARIMA models for DPM

| **Model Statistics** | **Stationary R-squared** | **RMSE** | **Normalized BIC** |  |
| --- | --- | --- | --- | --- |
|  |  |  |  |  |
| Holt | 0.59 | 0.47 | -1.51 |  |
| ARIMA(0,2,2) | 0.59 | 0.47 | -1.49 |  |
| ARIMA(1,2,1) | 0.57 | 0.48 | -1.44 |  |
| **ARIMA(1,1,1)*** | **0.64** | **0.50** | **-1.35** |  |

** -> Final Selected Model*

**Step 5: Model Diagnosis**

The diagnostic assessment of the model is an important and integral component of model building. Thus, we made the residual plots of ACF and PACF of selected final models to assess model adequacy. The random uneven variation around 0 indicate that SARIMA (3,2,1)(1,0,1) and ARIMA (1,1,1) models for CPM and DPM, respectively are adequate.

| 4(a): Diagnostic ACF and PACF plot for CPM | 4(b): Diagnostic ACF and PACF plot for DPM |
| --- | --- |
| 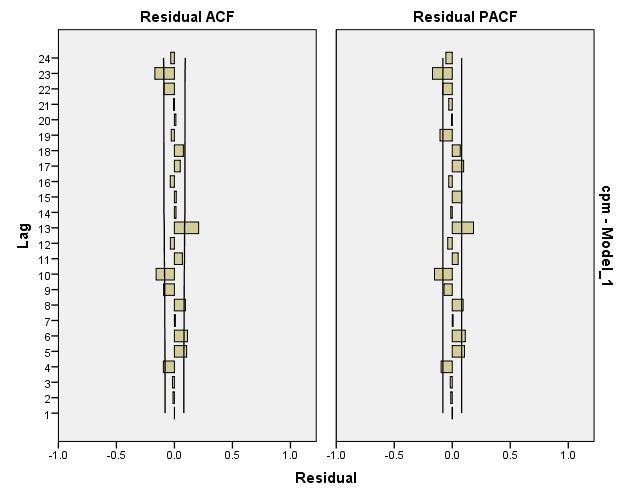 | 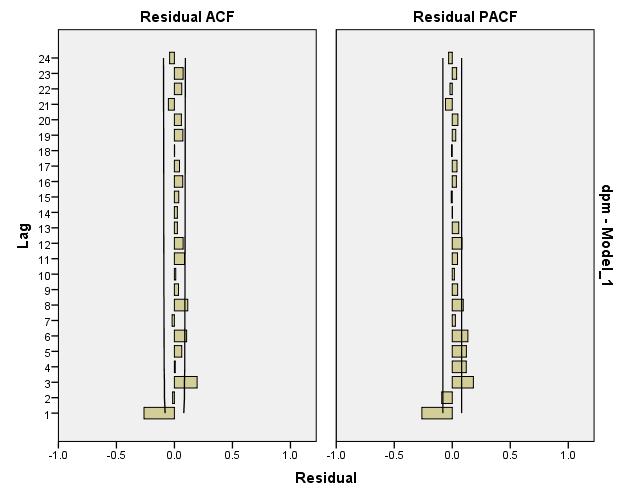 |
